# Energy Absorption and Exposure Buildup Factors of Essential Amino Acids

**DOI:** 10.1155/2014/359754

**Published:** 2014-01-29

**Authors:** Ertuğrul Bursalıoğlu, Begüm Balkan, H. Birtan Kavanoz, Mustafa Okutan, Orhan İçelli, Zeynel Yalçın

**Affiliations:** Department of Physics, Faculty of Arts and Sciences, Yıldız Technical University, Davutpasa, 34220 Istanbul, Turkey

## Abstract

The effective atomic number and effective electron density in amino acids are of significant interest due to their use in various applications. The energy absorption buildup factors, exposure buildup factors, effective atomic numbers, and electron densities of essential amino acids such as Leucine (C_6_H_13_NO_2_), Lysine (C_6_H_14_N_2_O_2_), Methionine (C_5_H_11_NO_2_S), Phenylalanine (C_9_H_11_NO_2_), Threonine (C_4_H_9_NO_3_), Tryptophan (C_11_H_12_N_2_O_2_), Valine (C_5_H_11_NO_2_), Arginine (C_6_H_14_N_4_O_2_), and Histidine (C_6_H_9_N_3_O_2_) were determined theoretically in the energy range 0.015–15 MeV.

## 1. **Introduction**

The effective atomic number and effective electron density are the basic essential quantities used for determining the penetration of X-ray and gamma photons in matter. In recent years, there has been a growing interest in X-rays and gamma photons in biological and other important materials commonly used in industrial, biological, agricultural, and medical applications such as nuclear diagnostic, radiation protection, nuclear medicine, radiation dosimeter, and radiation biophysics. The energy absorption buildup factor (EABF) and exposure buildup factor (EBF) values prove to be useful in choosing a substitute composite material in place of an element for the required energy [1].

Constituting the largest living matter in all types of cells, the amino acids are the most abundant macromolecules that exist in living cells. The primary use of amino acids is that they are the building blocks of protein. Much as an entire train that is made up of different carriages, so too are proteins made up of amino acids.

Some proteins contain as few as three amino acids, while others can contain hundreds. The human body is constructed of 20 different amino acids. Out of the 20 amino acids, human can produce 12 of them, which are called nonessential amino acids. The other eight are defined as essential amino acids which must be supplied through food consumption.

The study has been carried out for some amino acids [2]. The frequent and vital applications of radiation and its sources in medical and biological field require detailed knowledge of effective atomic numbers and effective electron densities of amino acids. Investigation of radiation effects on biologically important molecules has the potential to offer insights into applications in medical physics and radiation biology fields [3].

Exposure buildup factor and energy absorption buildup factor have been determined for human organs and tissues [4]. For this reason, there is a need for gamma-ray buildup factors of essential amino acids in diagnostics, dosimeter, and radiation therapy for absorbed dose estimations. This prompted us to conduct the study on the buildup of photons in some essential amino acids for which the buildup factor data cannot be found in any compilation or tabulation. It is believed that with proper knowledge of buildup factors of essential amino acids, energy absorption can be carefully controlled. Therefore, the results of the present paper will help in estimating safe dose levels for radiotherapy.

## 2. **Materials and Methods**

The effective atomic number *Z*_eff_ for any nonessential amino acid is given by

(1) $$\begin{array}{c} Z_{\text{eff}}=\frac{\sigma_{a}}{\sigma_{e}}, \end{array}$$

where *σ*_*a*_ and *σ*_*e*_ are the effective (average) atomic and total effective electronic cross-sections, respectively. The effective electron density, *N*_el_ (number of electrons per unit mass), can be derived by using

(2) $$\begin{array}{c} N_{\text{el}}=\frac{{\left(\mu /\rho \right)}_{c}}{\sigma_{e}}=\frac{N_{A}}{M}Z_{\text{eff}}\sum_{i}n_{i}. \end{array}$$

The photon atomic parameters such as total mass attenuation coefficients, effective atomic, molecular, and electronic cross-sections, and effective atomic numbers for nonessential amino acids are presented in detail in the study conducted by Baştuğ et al. [5]. The molecular formula and weight of essential amino acids are given in Table 1. Buildup factor is an important parameter in distribution of photon flux in every object. Experiments are carried out to achieve gamma-ray buildup factors which are not easy to obtain in general. Therefore, studies of gamma-ray buildup factors have been conducted using some calculations. Buildup factor is defined as the ratio of the total detector response to that of unscattered photons. Buildup factor data is the basic requirement for point kernel calculations commonly used in shield design. It has been classified into two categories called energy absorption buildup factor (EABF) and exposure buildup factor (EBF). The EABF is the buildup factor in which the quantity of interest is the absorbed or deposited energy in the interacting material and the detector response function is that of absorption in the interacting material. For the EBF, the quantity of interest is the exposure whereas the detector response function corresponds to that of the absorption in the air. This means there is an assumption that exposure is equivalent to the absorbed dose in air as measured by the nonperturbing detector. The G-P fitting method has been used by different researchers for studying different solvents, polymers, human teeth, and tissue substitute materials, respectively [6–8].

We have not calculated the G-P fitting parameters, but we have developed a new interpolate method instead. This method has been used to determine parameters for some thick oxide films by Kavanoz et al. [9]. In this method, we have calculated the values of *Z*_eff_ by means of WinXCOM for some elements adjacent to *Z*_eff_. Then, we have considered the calculated effective atomic number of our samples to compare G-P fitting parameters available in ANSI/ANS6.4.3 standard data for some specific element within a specific energy range as well as two elements that satisfy *Z*_1_ < *Z*_eff_ < *Z*_2_ condition. This process has allowed us to find the fitted values of EABF and EBF that correspond to our samples. That is to say, after calculating EABF and EBF by using G-P fitting parameters of the elements with *Z*_1_ and *Z*_2_ atomic numbers for the energy in question, we have obtained EABF and EBF values of corresponding Z_eff_ by interpolation.

## 3. **Results and Discussion**

**Figure 1:**
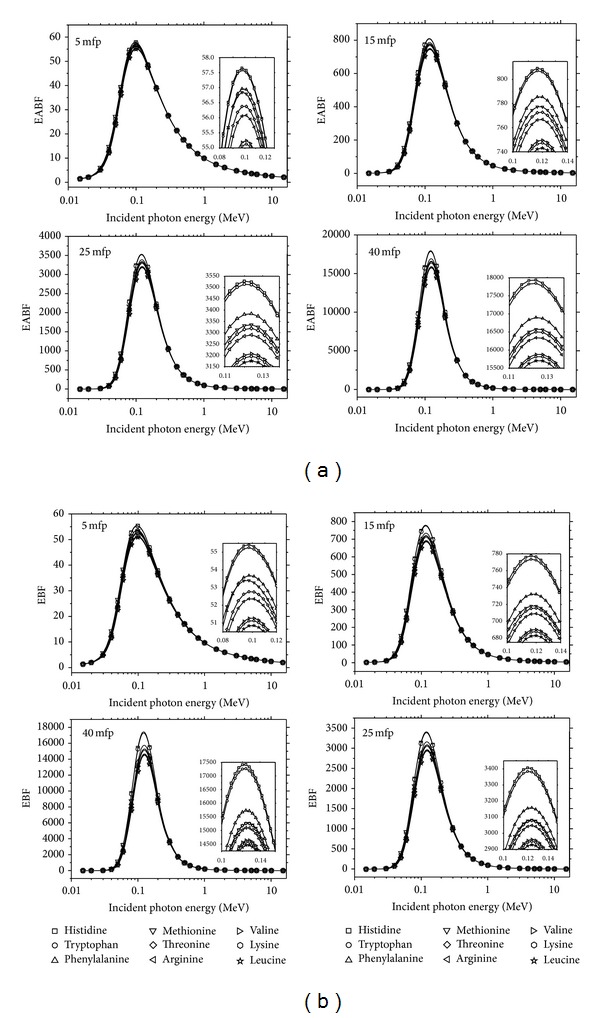
(a) Variation of EABF with incident photon energy for essential amino acids at selected penetration depths (5–40 mfp). (b) Variation of EBF with incident photon energy for essential amino acids at selected penetration depths (5–40 mfp).

**Figure 2:**
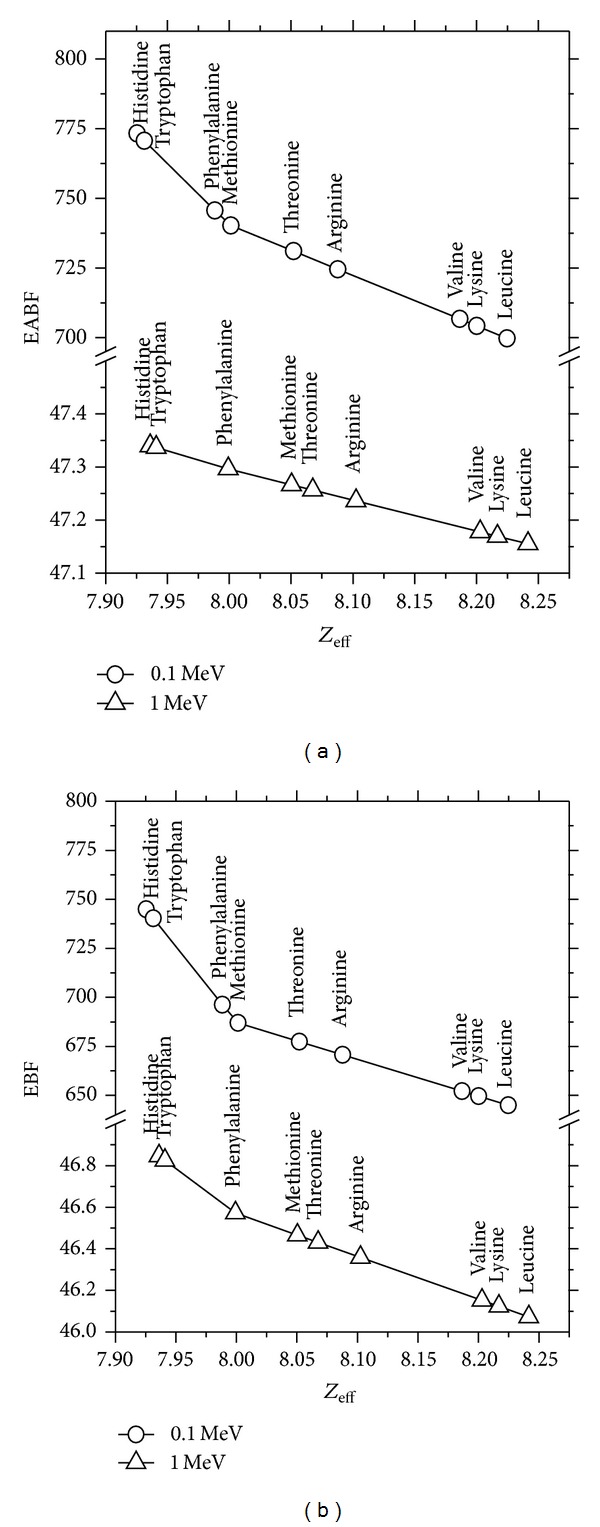
Variation of EABF and EBF with *Z*_eff_ for essential amino acids at selected energy (0.1 and 1 MeV).

**Figure 3:**
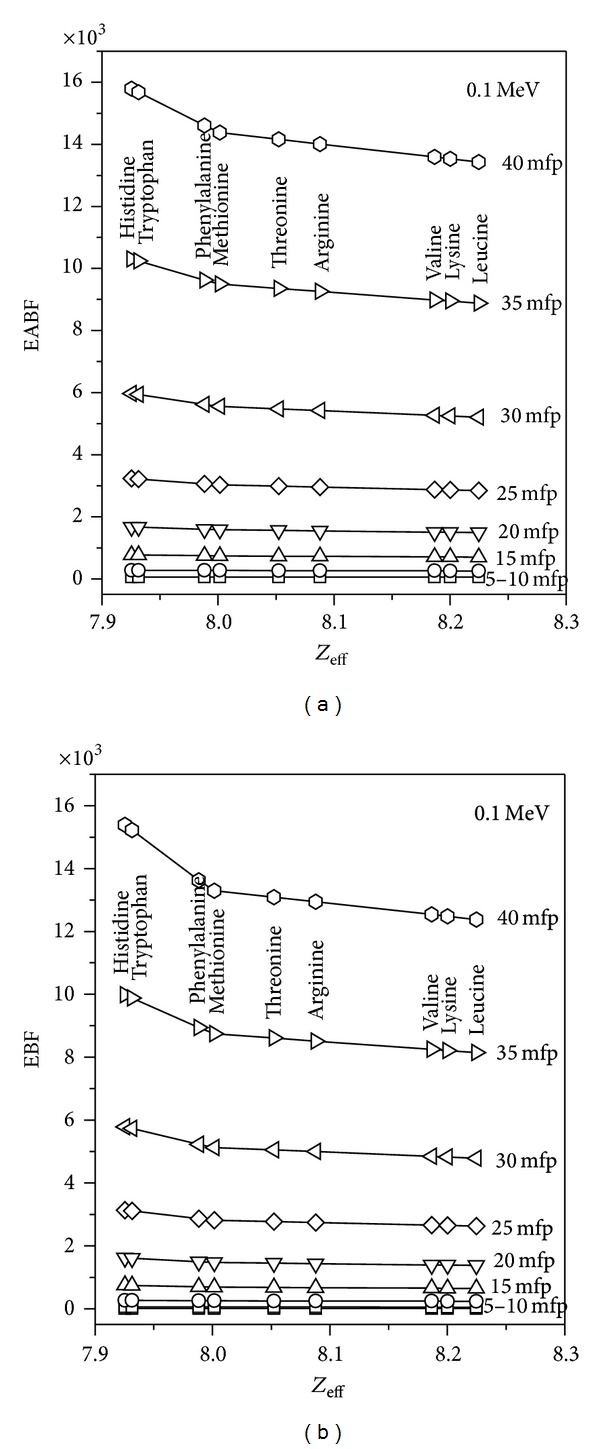
Variation of EABF × 10^3^ and EBF × 10^3^ with *Z*_eff_ for essential amino acids at selected energy (0.1 MeV).

**Figure 4:**
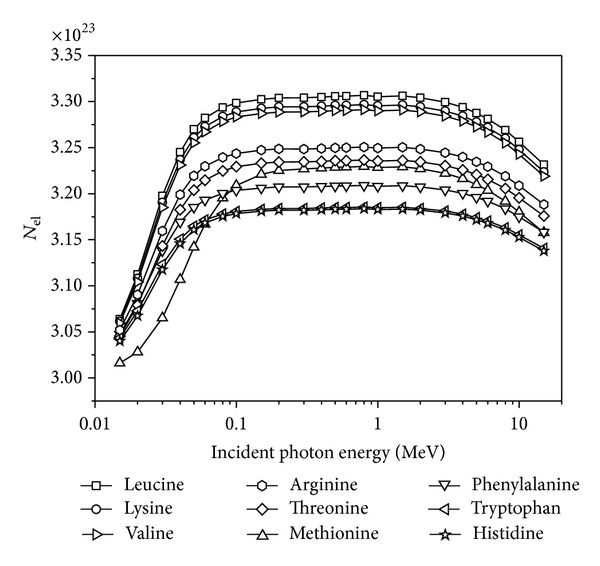
The dependence of *N*_el_ on photon energy.

We will discuss how the buildup factors vary with incident photon energy, chemical composition, and effective atomic number in the following paragraphs. As seen in Figures 1(a) and 1(b), the energy absorption buildup factors (EABF) and exposure buildup factors (EBF) of essential amino acids are compared with penetration depths of 5, 15, 25, and 40 mfp, respectively. It has been observed that the EABF and EBF values are generally the highest for Histidine and lowest for Leucine in the energy range 0.015–15 MeV. This state has been confirmed by V. A. Beatty and W. J. Beatty [10]. This is also indicated by Leucine which has a high *Z*_eff_, while Histidine has a low *Z*_eff_. Similar results are observed at 5, 15, 25, and 40 mfp. For this reason, it can be concluded that EABF and EBF depend on chemical composition. In other words, EABF and EBF values decrease with the increasing *Z*_eff_ values of essential amino acids for the selected range. Similar results are observed at different penetration depths. As seen in Figure 2, EABF and EBF are a function of *Z*_eff_ for 15 mfp at 0.1 and 1 MeV energy. It is seen that EABF and EBF decrease remarkably with increasing *Z*_eff_. This relationship is mainly due to the presence of molecular weight. Similar trends are observed in other mfp and energy. As a result, smaller EABF and EBF can be associated with materials having higher *Z*_eff_ which is in the range 7-8 for essential amino acids. Manohara et al. (2010) have determined EABF for thermoluminescent dosimetry materials commonly used for the construction of tissue such as LiF, BeO, and Li_2_B_4_O_7_ whose *Z*_eff_ is relevant (6.66–8.24) [11]. This is remarkable because of our *Z*_eff_ range of essential amino acids which is in between 7.50 and 8.20.  Figure 3 demonstrates the variation of EABF and EBF with *Z*_eff_ for all penetration depths of 0.1 MeV energy. It has been observed that EABF and EBF increase remarkably with increasing mfp. This trend is mainly due to the *Z*_eff_ of the material that should match as closely as possible with that of irradiated essential amino acids.  Figure 4 shows the variation of *N*_el_ for all essential amino acids in the energy range 0.015–15 MeV. It is seen that the lowest values of *N*_el_ belong to methionine up to 0.06 MeV. Yet, above 0.06 MeV, Histidine has the lowest values of *N*_el_. It is observed that for all energy ranges, Leucine has the largest *N*_el_ value. As a result, Leucine has the lowest EABF and EBF values when compared to other essential amino acids. This situation may be attributed to small electron density which can appear in the form of carbon-rich or low density and oxygen-rich or higher density.

Elevated electron density increases the density of tumor, which explains the macroscopic hard consistency of the tumor [12]. Also Kubota et al. have tried to establish a correlation in order to correlate between amino acid profiles and diagnostically with kinds of malignant tumors [13]. Therefore, it is very important to measure photon atomic parameters such as effective atomic number and effective electron densities (electron/g) of selected essential amino acids. Many researchers have studied the effective electron densities of different materials in wide energy ranges [14–17].

In this study, the relationship between histology of essential amino acids and their obtained electron densities is discussed. *N*_el_ is calculated by means of *Z*_eff_ and this value of *N*_el_ of the amino acids can be used as a device for the diagnosis of tumor.

The novelty of the work is that the essential amino acids have been investigated using the mass attenuation coefficients, the effective atomic numbers, the effective electron densities, the energy absorption (EABF), and exposure (EBF) buildup factors in the energy range 0.015 MeV–15 MeV on the basis of the mixture rule. The effective atomic number *Z*_eff_ and the corresponding effective electron density *N*_el_ of some essential amino acids have been calculated in the energy range 0.015–15 MeV. The value of effective atomic number for essential amino acids containing H, C, N, and O elements is almost constant in the energy range 5–1500 keV [3]. We have confirmed this situation. It should be noted that the *Z*_eff_ and *N*_el_ are based on an underlying theory of X-ray and gamma-ray interactions. It is expected that the new results on *Z*_eff_, *N*_el_, EABF, EBF, and other photon atomic parameters of essential amino acids presented here will be useful due to their importance in medical dosimeter. To the best of our knowledge, the results reported are the first of their kind and have not been reported previously for selected energy range.

The most important result of this study is that there is a direct relationship for the sensor between gamma-rays and the electron density or number of electron. Moreover, we hope that assigned atomic parameters may be used as a guide to prepare amino acid biosensor [18]. A companion parameter to the *Z*_eff_ of particular interest in medical and biological applications is the *N*_el_, which is used for computing the energy deposition by photons (X-ray, gamma-ray) at a site (a “volume of interest”) in biological, shielding, and other materials.

Finally, Leucine and lysine show the best sensing properties towards the gamma-rays in energy range 0.015–15 MeV. Significant variations in effective electron density values of Leucine in energy range 0.015–15 MeV make it the best sensor for gamma-rays in the selected energy range.

Bianchi and Díez-Sampedro demonstrate that HSGLT3 functions as a sugar sensor in vivo and that mutating a single amino acid converts this sugar sensor into a sugar transporter similar to SGLT1 [19].

## 4. **Conclusion**

From the present investigation, the following points have been concluded.

- Leucine shows significant variations in electron densities in the selected energy range; hence, it will work as the best gamma-ray sensor in the selected energy range [20].
- The electrical conductivity may be determined, theoretically, by means of effective electron density *N*_el_. This parameter shows amino acid biosensor for the essential amino acids. Determination of the electron density may shed light on the existence of tumor, which might then be used in radiology (diagnostics) and in radiotherapy (treatment of tumor).
- The chemical compositions play an important role in the buildup of gamma photons within the selected amino acids. In energy range 0.015–0.1 MeV, energy absorption buildup factor increases with increasing *Z*_eff_. In energy range 0.1–0.8 MeV, energy absorption buildup factor decreases with increasing *Z*_eff_. In energy range 0.8–15 MeV, energy absorption buildup factor decreases with decreasing *Z*_eff_.

From the present investigation, the most important following point has been concluded: the treatment consists of a tightly controlled intake of foodstuffs, the consumption of a tailor-made blend of amino acids, often designed specifically for the type of cancer that is being battled, and a defined regimen of nutritional supplements, with an addendum of those that must be avoided [21]. Also, it should be noted that the causes of cancer and a variety of disease aspects. It may be possible an interest of the electron density and food consumption. Summarize the correlation between the electron density and food consumption is thought.

The effective atomic number, effective electron density, energy absorption buildup factor, and exposure buildup factor have shed a new light on the underlying radiation physics and thus the findings of this study will be useful in radiation therapy, diagnostics, dosimeter, and medical and biological applications. With proper knowledge of buildup of photons in essential amino acids, energy absorption in proteins can be carefully controlled.

## Highlights

- The photon atomic parameters calculated for the essential amino acids.
- The direct relationship between gamma-rays and the electron density or number of electron.
- Determination of the electron density may shed light on the existence of tumor.

## Figures and Tables

**Table 1:** The molecular formula and weight of essential amino acids.

Name	Molecular formula	Molecular weight (g/mol)
Leucine	C_6_H_13_NO_2_	131.17
Lysine	C_6_H_14_N_2_O_2_	146.19
Methionine	C_5_H_11_NO_2_S	149.21
Phenylalanine	C_9_H_11_NO_2_	165.19
Threonine	C_4_H_9_NO_3_	119.12
Tryptophan	C_11_H_12_N_2_O_2_	204.23
Valine	C_5_H_11_NO_2_	117.15
Arginine	C_6_H_14_N_4_O_2_	174.20
Histidine	C_6_H_9_N_3_O_2_	155.16

## References

[1] Han İ, Demir L (2009). Studies on effective atomic numbers, electron densities from mass attenuation coefficients in Ti_x_Co_1-x_ and Co_x_Cu_1-x_ alloys. *Nuclear Instruments and Methods in Physics Research B*.

[2] Turşucu A, Önder P, Demir D, Özünlüer T (2013). Studies on mass attenuation coefficient, effective atomic number and electron density of some amino acids. *International Journal of Physical Sciences*.

[3] Pawar PP, Bichile GK (2011). Molar extinction coefficients of some proteins. *Archives of Physics Research*.

[4] Manohara SR, Hanagodimath SM, Gerward L (2011). Energy absorption buildup factors of human organs and tissues at energies and penetration depths relevant for radiotherapy and diagnostics. *Journal of Applied Clinical Medical Physics*.

[5] Baştuğ A, İçelli O, Gürol A, Şahin Y (2011). Photon energy absorption parameters for composite mixtures with boron compounds. *Annals of Nuclear Energy*.

[6] Singh PS, Singh T, Kaur P (2008). Variation of energy absorption buildup factors with incident photon energy and penetration depth for some commonly used solvents. *Annals of Nuclear Energy*.

[7] Singh T, Kumar N, Singh PS (2009). Chemical composition dependence of exposure buildup factors for some polymers. *Annals of Nuclear Energy*.

[8] Kurudirek M, Topcuoglu S (2011). Investigation of human teeth with respect to the photon interaction, energy absorption and buildup factor. *Nuclear Instruments and Methods in Physics Research B*.

[9] Kavanoz HB, Yağcı Ö, Yalçın Z (2014). Photon parameters for g-rays sensing properties of some thick oxide films. *Vacuum*.

[10] Beatty VA, Beatty WJ (1963). *Radiation Recovery Enhanced through Inhibitors of Protein Synthesis and Amino Acids*.

[11] Manohara SR, Hanagodimath SM, Gerward L (2010). Energy absorption buildup factors for thermoluminescent dosimetric materials and their tissue equivalence. *Radiation Physics and Chemistry*.

[12] Antoniassi M, Conceição ALC, Poletti ME (2012). Study of electron densities of normal and neoplastic human breast tissues by Compton scattering using synchrotron radiation. *Applied Radiation and Isotopes*.

[13] Kubota A, Meguid MM, Hitch DC (1992). Amino acid profiles correlate diagnostically with organ site in three kinds of malignant tumors. *Cancer*.

[14] Prasad SG, Parthasaradhi K, Bloomer WD (1998). Effective atomic numbers for photoabsorption in alloys in the energy region of absorption edges. *Radiation Physics and Chemistry*.

[15] Manohara SR, Hanagodimath SM, Thind KS, Gerward L (2008). On the effective atomic number and electron density: a comprehensive set of formulas for all types of materials and energies above 1 keV. *Nuclear Instruments and Methods in Physics Research B*.

[16] Kurudirek M, Aygun M, Erzeneoğlu SZ (2010). Chemical composition, effective atomic number and electron density study of trommel sieve waste (TSW), Portland cement, lime, pointing and their admixtures with TSW in different proportions. *Applied Radiation and Isotopes*.

[17] Damla N, Baltas H, Çelik A, Kiriş E, Çevik U (2012). Calculation of radiation attenuation coefficients, effective atomic numbers and electron densities for some building materials. *Radiation Protection Dosimetry*.

[18] Turmanova S, Trifonov A, Kalaijiev O, Kostov G (1997). Radiation grafting of acrylic acid onto polytetrafluoroethylene films for glucose oxidase immobilization and its application in membrane biosensor. *Journal of Membrane Science*.

[19] Bianchi L, Díez-Sampedro A (2010). A single amino acid change converts the sugar sensor SGLT3 into a sugar transporter. *PLoS ONE*.

[20] Conigrave AD, Franks AH, Brown EM, Quinn SJ (2002). L-amino acid sensing by the calcium-sensing receptor: a general mechanism for coupling protein and calcium metabolism?. *European Journal of Clinical Nutrition*.

[21] Brennan MF (1981). Total parenteral nutrition in the cancer patient. *The New England Journal of Medicine*.

